# A Case Report of Eosinophilic Esophagitis Accompanying Hypereosinophilic Syndrome

**DOI:** 10.1155/2012/683572

**Published:** 2012-08-01

**Authors:** Mahreema Jawairia, Ghulamullah Shahzad, Jaspreet Singh, Kaleem Rizvon, Paul Mustacchia

**Affiliations:** Department of Medicine, Nassau University Medical Center, 2201 Hempstead Turnpike, East Meadow, NY 11554, USA

## Abstract

Hypereosinophilic syndrome is a blood disorder characterized by the overproduction of eosinophils in the bone marrow with persistent peripheral eosinophilia, associated with organ damage by the release of eosinophilic mediators. Although HES can involve multiple organ systems, GI tract involvement is very rare. Few cases of HES presenting with gastritis or enteritis have been reported worldwide. To date, HES presenting with esophagus involvement has only been reported once. Here, we present a 39-year-old Hispanic female patient with history of HES presenting with complaints of dysphagia and generalized pruritus.

## 1. Introduction

Hypereosinophilic syndrome (HES) is a leukoproliferative disorder  marked  by  a sustained overproduction of eosinophils [1]. In addition to its eosinophilia, the uniqueness of the syndrome is its marked predilection to damage specific organs. History for allergic disorders, medications, and travelling should be sought, and patients should be investigated for helminthic/parasitic infections. HES is more common in men than women and tends to occur between the ages of 20 and 50, although few cases have been reported in children [1].

## 2. Case Report

A 39-year-old Hispanic female presented with complaints of generalized body itching and difficulty in swallowing to both solids and liquids for the past two years. Dysphagia was progressively worsening in severity for the past few weeks and was associated with nausea and vomiting. Patient denied any weight loss, diarrhea, hematochezia, melena, odynophagia, hematemesis, and abdominal pain. Past medical history included asthma and hypereosinophilic syndrome. She also denied any tobacco, alcohol, or illicit drug use. On physical examination, elbows, hands, and the soles of the feet were hyperkeratinized. Laboratory findings showed Hb/Hct of 13.3/39.5, WBC of 8.1, absolute neutrophilic count of 800/*μ*L (normal reference value: 1500–8000/*μ*L), and absolute eosinophil count was increased to 4000/*μ*L (normal: 0–450/*μ*L). Liver-related tests and connective tissue disorder tests were unremarkable.

**Figure 1:**
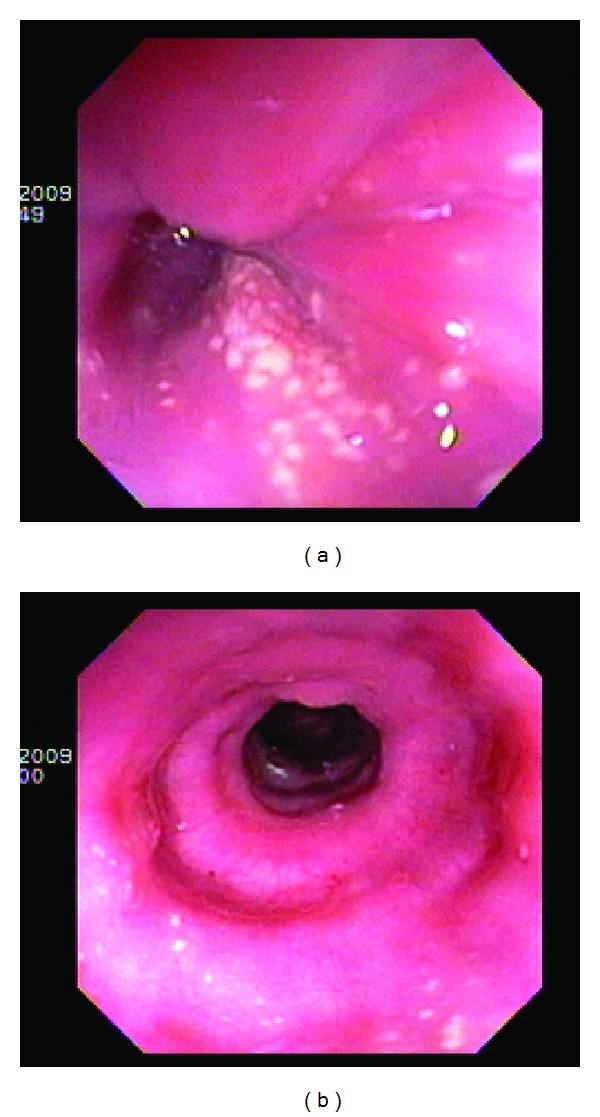
EGD showing diffuse pin-point whitish exudates and ringed.

Esophagogastroduodenoscopy (EGD) revealed whitish exudates noted in the esophagus (Figure 1) with normal stomach and duodenum. Brushings were negative for *Candida* species, and antral biopsy was negative for *Helicobacter pylori*. A Double Contrast Esophagram examination revealed abnormal peristalsis/motility with the presence of a questionable mild stricture in distal esophagus. The patient was started on diflucan and protonix 40 mg daily but returned to GI clinic four weeks later with persistent dysphagia.

**Figure 2:**
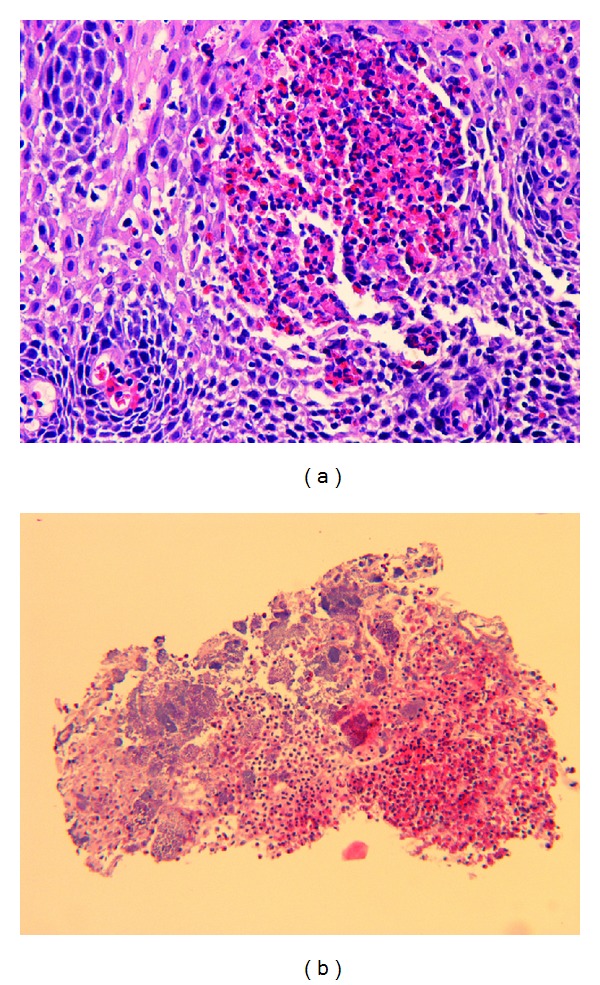
Biopsy revealing eosinophils in the esophagus.

EGD was repeated, and it revealed persistent whitish pin-point exudates in the mid and lower esophagus. Then, patient's protonix was increased to 40 mg twice daily and was told to followup in GI clinic in two weeks. On followup visit, her brushings showed no *Candida* and midesophagus biopsy showed eosinophilic infiltrate (Figure 2) and findings were consistent with acute eosinophilic esophagitis and microabscesses with eosinophil count of 65/HPF. Subsequently, she was started on fluticasone 40 mcg twice, daily and on a follow-up visit, she reported marked improvement in her dysphagia.

## 3. Discussion

HES is a severe and devastating multisystem disorder associated with considerable morbidity. It entails several heterogeneous disorders characterized by persistent blood eosinophilia and eosinophil-related end-organ damage with no distinguishable cause. In 1968, Hardy and Anderson [2] were the first ones to describe HES with persistent eosinophilia related to multiple tissue damage. Later Chusid et al. [3] described three characteristics required to diagnose HES, such as an unremitting absolute eosinophil count (AEC) greater than >1500/*μ*L for more than 6 months, no detectable etiology for eosinophilia (e.g., parasitic infection), and patients must have signs and symptoms of organ involvement. The organ systems most commonly affected in HES are the heart, nervous system, skin, lungs, and gastrointestinal tract [4].

Involvement of the heart, skin, nervous system, and lungs presents with fatigue, cough, breathlessness, muscle pains, angioedema, rash, and fever in about 40% to 64% of patients, whereas gastrointestinal and liver involvements are less common (14% to 32% each) [5]. Liver involvement may take the form of chronic active hepatitis, focal hepatic lesions, eosinophilic cholangitis, or the Budd-Chiari syndrome [6]. Gastrointestinal manifestations include eosinophilic gastritis, enteritis, and/or colitis causing weight loss, abdominal pain, vomiting, and/or severe diarrhea [1]. Our patient had all three of the diagnostic characteristics with involvement of the esophagus, which is a rare finding. The pharmacologic options for management of HES include tyrosine kinase inhibitors in those with 4q12 deletion and other drugs like glucocorticoids [7], interferon alpha [8], and chemotherapeutic agents, such as hydroxyurea [9]. Our patient responded very well to corticosteroid therapy and showed marked improvement in her symptoms. Since dysphagia is a very common presentation of eosinophilic esophagitis (EE), one might argue that this can be EE. However, there are findings which dispute against it. Peripheral eosinophilia can be seen in eosinophilic esophagitis, but it is almost always mild. Hence, it is imperative for the medical community to include HES as a differential diagnosis in a patient with refractory dysphagia not responding to PPI therapy.
